# Olfactory ensheathing cell transplantation alleviates cancer pain by inhibiting P2X7 receptor expression–mediated activation of microglia

**DOI:** 10.1097/JS9.0000000000002976

**Published:** 2025-07-09

**Authors:** Wen-Jun Zhang, Ji-Peng Liu, Yong-Sheng Xu, Cheng Zuo, Jun-Xiang Liao, Fu-Qi Zhu, Jia-Ling Hu

**Affiliations:** a Department of Rehabilitation Medicine, The Second Affiliated Hospital, Jiangxi Medical College, Nanchang University, Nanchang, Jiangxi, China; b The Second Affiliated Hospital, Jiangxi Medical College, Nanchang University, Nanchang, Jiangxi, China; c Department of Emergency Medical, The Second Affiliated Hospital, Jiangxi Medical College, Nanchang University, Nanchang, Jiangxi, China

**Keywords:** alleviate, cancer pain, OECs, P2X7R

## Abstract

Peripheral nerve invasion can occur during cancer progress, which seriously affects the patient’s quality of life and brings pain. There is currently no effective treatment method. Therefore, in this study, we established a model of breast cancer bone metastasis and transplanted olfactory sheath cells (OECs) into rats to explore a new cell therapy strategy for treating cancer pain. The results showed that compared with the cancer pain group and the vehicle group (DMEM/F12 medium solution), OEC transplantation improved tibial bone destruction and osteolytic lesions, increased the mechanical withdrawal threshold and thermal withdrawal latency, and alleviated cancer pain behavior in rats. *In vitro* experiments found that OECs inhibited microglia activation induced by P2X7 receptor (P2X7R) activator BzATP, reduced the expression of IL-1β and IL-18, and protected neuron survival. Further *in vivo* experiments showed that transplantation of OECs reduced the expression levels of P2X7R, NLRP3, apoptosis-associated spotted protein (ASC), and Caspase-1 in spinal cord tissue, reduced the concentration levels of IL-1β and IL-18 in rat serum, and promoted the growth of peripheral axons. Our conclusions suggest that OEC transplantation may alleviate cancer pain by downregulating P2X7R expression, inhibiting NLRP3/ASC/Caspase-1 signaling, improving neuroinflammatory responses, and thus alleviating cancer pain. These data indicate that OECs may become promising targets for treating cancer pain.

## Introduction

Patients with advanced cancer may experience severe or persistent pathological pain, which may compress or invade peripheral nerves during tumor growth and metastasis, lead to sensitization of peripheral sensory organs, and enhance pain information transmission^[1,2]^. Cancer pain models can simulate bone metastasis in primary bone tumors or other tumors such as breast cancer and prostate cancer, and enhanced activity of osteoclasts can increase the incidence of bone cancer pain^[3]^. In addition to stimulating and damaging related nerves during the onset of cancer pain, it can also activate immune cells and release a large number of pro-inflammatory cytokines (such as TNF-α, IL-1β, and IL-18), inducing inflammatory reactions and pain^[4,5]^. The manifestations of cancer pain are diverse and vary greatly. They may include one or more types of paresthesia, such as hypersensitivity, paraesthesia, and spontaneous pain^[6]^. Especially in the middle and advanced stages of cancer, the incidence of pain is high, and moderate to severe pain is the main cause^[7]^. However, the pain of most cancer patients is not effectively controlled, causing serious physical and mental harm to patients and leading to a significant decline in their quality of life. In addition, cancer pain can reduce physical function and disease resistance, resulting in reduced treatment effects. Therefore, the treatment of cancer pain brings greater clinical challenges. Drugs (such as opioids, non-opioids, and adjuvant analgesics) are usually used clinically to relieve pain. However, drug analgesia is not effective and lasts for a short time, and long-term use leads to greater dependence and side effects. Therefore, finding an effective treatment for cancer pain has become an urgent problem.

**HIGHLIGHTS**

- For the first time, we have found that olfactory ensheathing cell transplantation has a functional effect on inhibiting breast cancer-induced bone destruction.
- For the first time, it was discovered that olfactory ensheathing cells can inhibit the therapeutic function of cancer-induced pain.
- Olfactory ensheathing cell transplantation may inhibit microglia activation by downregulating P2X7 receptor expression, thereby producing a possible mechanism for analgesia.

Studies have confirmed that P2X7 receptor (P2X7R) plays an important role in cancer progression and pain information transmission, becoming a potential target for exploring pain and cancer treatment^[8]^. Activation of P2X7R can activate microglia and promote the release of inflammatory cytokines, mediate cell death/apoptosis, produce neuronal toxic effects, sensitize peripheral receptors, enhance sensory information transmission, and induce pain^[8–10]^. The key role of P2X7R in mediating the inflammatory response is to activate the P2X7R/nucleotide binding domain, leucine-rich family, and NLRP3 inflammasome pathway, which leads to the release of IL-1 family cytokines, thereby inducing cell death^[11,12]^. NLRP3 inflammasome complex includes NLRP3, a NOD-like receptor that is a sensor of inflammasome activation, an apoptosis-associated spotted protein (ASC), and caspase-1 (inflammatory protease caspase-1). Activation of NLRP3 leads to the maturation of caspase-1, which is subsequently implicated in the cleavage of IL-1β and IL-18 into biologically active cytokines^[13,14]^. In the cancer pain model, the increased ATP levels in rat cerebrospinal fluid activate P2X7R in spinal microglia, increase the expression levels of phosphorylated p38 and IL-18, and produce pain. Inhibition of the P2X7R/p-38/IL-18 pathway can inhibit neuronal overactivity and reduce pain in advanced cancer^[15]^. These studies reveal that targeting the P2X7R can serve as another interesting potential therapeutic target for cancer pain treatment.

With continuous research and exploration in recent years, people have introduced the concept of cell transplantation, which transplants functionally active cells into a host to produce analgesic pharmacological properties. Cells transplanted into the host can continuously secrete multiple active nutritional factors to relieve pain. These active cells transplanted into the body can reduce hyperalgesia or reduce pain threshold in the host^[16,17]^. Olfactory sheath cells (OECs) are a special type of glial cells that can continuously update and survive in the nervous system and have strong supporting functions in nerve regeneration. OECs can secrete multiple active nutritional factors, such as neurotrophic factor 3, neurotrophic factor 4, nerve growth factor, ciliary nerve growth factor, extracellular matrix components, and cell adhesion molecules, to promote axon regeneration and myelination^[18,19]^. In addition, OECs can also improve local inflammatory responses after nerve injury, reduce the release of pro-inflammatory cytokines, change the inflammatory microenvironment around the injured local tissue, and have the effect of protecting and repairing injured nerves^[19,20]^. Studies have found that OEC transplantation increases the number of regenerated neurons in rats with facial nerve transection injury, improves the shape of nerve fibers, increases the number of myelinated nerve fibers, nerve fiber diameter, and myelination, and promotes regeneration and functional recovery after facial nerve injury in rats^[18]^. Microencapsulated OEC transplantation reduces P2X7R expression in L4-5 dorsal root ganglia and alleviates neuropathic pain in rats^[21]^. Transplantation of OECs into the trigeminal nerve injury in rats reduced the expression of P2X7R in the trigeminal ganglia and effectively alleviated pain behavior in rats^[22]^.

These studies conducted a preliminary discussion on the downregulation of P2X7R and behavioral phenotypes by OEC transplantation, but did not study the possible mechanism of OECs regulating P2X7R and their mediated downstream signals, and regulating the influence of microglia on pain behavior. These studies demonstrate the functional role of OECs in promoting nerve regeneration and the therapeutic effect of relieving pain (neuropathic pain and trigeminal pain types), indicating the therapeutic potential of OECs in treating pain. However, there is currently no report on whether OECs affect pain caused by bone metastases from breast cancer. Therefore, for the first time, we investigated the therapeutic effect and possible molecular mechanisms of OECs on cancer pain. We investigated the effect of OECs on bone destruction induced by bone metastases in breast cancer and their potential in treating pain induced by bone metastases. Furthermore, the protective effect of OECs on neurons and their mechanism on P2X7R-induced microglia-mediated inflammatory response were further studied through *in vivo* and *in vitro* experiments. Therefore, this study provides a new treatment strategy and theoretical basis for the future treatment of cancer pain.

## Materials and methods

### Culture and identification of OECs

OECs were cultured and purified (the method of different rates of cell attachment). The neonatal SD rat olfactory bulb tissue was taken and placed it in 0.01 M PBS buffer at 4°C and washed it three times. The tissue was cut into powder with sterilized ophthalmic scissors and thoroughly grind the shredded tissue with a grinding stick. 0.25% trypsin was added and digested at room temperature for 10 min, and then filtered with a sterile 200-mesh cell sieve to filter out undigested tissue fragments. Dulbecco’s Modified Eagle’s/Ham’s F12 medium containing 15% fetal bovine serum was added to terminate the digestion and centrifuged (1000 rpm/min, 5 min), and the liquid in the centrifuge tube was poured away. After the cell concentration was diluted with 1:1 Dulbecco’s Modified Eagle’s/Ham’s F12 medium containing 15% fetal calf serum and 1% penicillin-streptomycin mixture to 1 × 10^9^/L, placed the culture bottle into a CO_2_ dressing box for culture. Then, the medium was changed every 1–2 days. Cell purity was then determined after day 9.

The OECs suspension on the ninth day of culture was digested with trypsin and seeded into a 24-well plate covered with PLL-coated coverslip. After the cells were crawling around, they were fixed with 4% paraformaldehyde. 0.3% hydrogen peroxide was added to block endogenous peroxidase for 10 min and washed with PBS. 0.1% Thiton X-100 was added and acted for 10 min and washed with PBS. 3% normal goat serum was added and blocked for 15 min without washing. Diluted rabbit primary antibody NGFRp75 (1:200, Wuhan Boshide Company) was added and reacted overnight at 4°C. The next day, washed with PBS, added diluted secondary antibody CY3-goat anti-rabbit IgG (1:400, Wuhan Boshide Company), reacted for 1 h at room temperature, and washed with PBS. DAPI fluorescent dye was added dropwise and, after 5 min of reaction, was washed with PBS, and about 20 μL of anti-fluorescence quenching and mounting solution was added to the sample, covered with the slide, and observed under a fluorescence microscope. The proportion of OECs that had been purified and identified reaches more than 95%.

### Cell culture

Breast cancer cell line Walker-256 (an undifferentiated, highly malignant, fast-growing, and highly invasive tumor cell: it is often used to study tumor growth and metastasis, especially bone metastasis) and BV2 microglia (Cell Bank of China Academy of Sciences, Shanghai, China) were cultured in DMEM medium containing 10% FSB (Dr DE Biological, Wuhan, China). The Petri dish was placed in a CO_2_ incubator for cultivation, the cell culture medium was changed every two to three days, and observed cell growth under a microscope.

### Establishment of rat tibial cancer pain animal model and rat groups

Thirty-two SD rats (both sexes weighing 150–200 g and aged 7–8 weeks: all animals live in a constant environment at 24°C; animals had enough food and water and were fed in a 12-h dark and 12-h light cycle) were randomly divided into four groups: sham group, cancer pain group, vehicle group, and OEC group, eight rats in each group. After anesthesia, the rats in each group were shaved, the upper segment of the tibia was cut open to expose the bone surface, and a dental drill was used to punch holes in the tibia. On postoperative day 0, the sham group was injected with normal saline. The remaining three groups were inoculated with Walker-256 breast cancer cells in the bone marrow cavity of the tibia of the right hind limb^[23–26]^. The breast cancer cell density was 1 × 10^6^/animal, 10 μL/animal. Then the drill holes were sealed with bone wax, the wounds were sutured, and penicillin was applied to the wounds to prevent infection. On day 0 after modeling, the OECgroup and the vehicle group were injected intrathecally with OECs and medium solution, respectively, with a cell density of 4 × 10^5^/animal and 40 μL/animal (100 000 cells/μL). The work has been reported in accordance with the ARRIVE guidelines (Animals in Research: Reporting In Vivo Experiments)^[27]^.

### Behavioral methods were used to detect pain changes in rats

#### Detection of MWL

Rats in each group were placed in a plexiglass box on days 0, 4, 8, 12, and 16 after surgery, and the mechanical withdrawal threshold (MWT) of experimental injury to the hind paw of rats on the same side was measured using VonFrey filaments. Started from the minimum bending force (0.008 g), repeated the test for 10 times on each filament, with the interval between each stimulation being at least greater than 15 s, and started the test after the reactions caused by stimulation (such as leg shaking and foot licking) completely disappear. Until the number of foot retraction reflexes occurs is greater than 5/10. Three times were repeated, and the average value was taken.

#### Detection of TWL

Rats in each group were placed in a plexiglass box on days 0, 4, 8, 12, and 16 after surgery, and irradiated with a BME-410C fully automatic thermal pain stimulator until the rear feet of the irradiated rats appeared to lift their legs to avoid the latent time of the heat-shrinking foot reflex. In order to prevent burns, the time was interrupted for 30 s and repeated three times, and the average value was taken.

### Neuron culture and identification

Spinal cord tissue of SD rats was taken, the tissue was cut with ophthalmic scissors, digested with 0.25% trypsin at 37°C for 30 min, and the digestion was terminated with fetal bovine serum. The cell suspension was collected and filtered with a 200-mesh screen. The cells were purified by density gradient centrifugation and cultured in DMEM medium +10% fetal bovine serum. After 24 h of incubation, cells could be used for identification.

The slides were removed, fixed them with 4% paraformaldehyde, and washed them with PBS. 0.1% TritonX-100 was added to soak for 10 min and rinsed with PBS. Goat serum blocking solution was incubated at room temperature for 20 min without washing. Rabbit anti-mouse NSE (neuron-specific antibody) polyclonal primary antibody (1:20, Sigma company) was added and reacted overnight at 4°C. The next day, rinsed with PBS, added with goat anti-rabbit fluorescent secondary antibody IgG (1:100, Sigma company), incubated at 37°C for 60 min, and rinsed with PBS. DAPI was stained for 2 min, then anti-fluorescent dye was added dropwise to seal, and the labeled cells were observed under a fluorescent microscope.

### Immunohistochemical staining

Rabbit primary antibody P2X7R (1:200, Abcam plc, Cambridge) and goat anti-rabbit IgG secondary antibody (1:1000, Sigma company) were subjected to avidin–biotin–horseradish peroxidase immunohistochemical reactions, respectively, and DAB was colored. The changes of P2X7R in the spinal cord were then observed. Four image fields of immunohistochemical reaction results were randomly selected, and the percentage of P2X7R-labeled positive cells was counted and analyzed using the Image J-Pro Plus 6.0 image analysis system.

### qRT-PCR

Twenty-four hours after OEC treatment of activated microglia, total RNA from microglia was extracted using the TRIzol kit (Trans, Beijing, China), and then reverse transcribed into DNA using the TagMan mRNA reverse transcription kit (Wuhan Boshide Company, China) for PCR. Subsequently, DNA was used as a template, and a qPCR kit was used to configure a 20-µL system for PCR reactions (reaction conditions: 95°C 30 s, 95°C 35 s, 60°C 1 min, 95°C 15 s, 40 reaction cycles). Three wells were set up for each sample, using β-actin as the internal reference. The expression level of P2X7R mRNA in each group was determined by the 2^−ΔΔCT^ method.

P2X7R primer:

Forward: 5′ CTGTGAAATCTTTGCCTGGTG 3′

Reverse: 5′TGTTTCTCGTAGTATAGTTGTGGC3′

β-actin primers:

Forward: 5′-TAAAGA CCTCTATGCCAACACAGT-3ʹ

Reverse: 5′-CACGATGGAGGGGCCGGACTCATC-3ʹ

### Western-blotting testing

On day 12 after surgery, total protein was extracted from the spinal cord tissue of rats in each group. Subsequently, the glass plate used for gel was washed with water to prepare the separation gel and the concentrated gel, respectively. The above separating glue was added to about 3/5 of the glass plate, and then added water to flatten the glue surface for about 30–50 min. When the separating glue solidifies, poured out the water on the glass plate, added concentrated glue to the glass plate, inserted a clean comb vertically into the glass plate, and let it stand at room temperature for 30–60 min. Then the comb was pulled out, removed the prepared glue from the glass plate, and placed it into an electrophoresis tank filled with 1xTEA (Dr DE Biological, Wuhan, China). Added 5 μL of Marker to the first well, and then added 10 μL of sample to the other wells in turn according to each group sequence. Subsequently, electrophoresis was performed for approximately 90 min (voltage 90 V). After the electrophoresis was completed, an NC membrane with a suitable size for the colloid was cut and transferred to the NC membrane (Dr DE Biological, Wuhan, China). The membrane was taken out from the electrophoresis tank and placed in a 5% skim milk powder for 60 min. The NC membrane was placed in the prepared primary antibody rabbit anti-mouse P2X7R (1:500, Abcam plc, Cambridge), rabbit anti-mouse NLRP3 (1:500, Sigma-Aldrich), rabbit anti-mouse ASC (1:1000, Wuhan Boshide Technology Co., Ltd.), Caspase-1 (1:1000, Wuhan Boshide Technology Co., Ltd.), and β-actin (1:1000, Wuhan Boshide Company) at 4°C for incubation overnight, and the membranes were washed with TBST for 10 min and repeated three times. Secondary goat anti-rabbit IgG antibody (1:5000, Wuhan Boshide, China) was added and shaken for 1 h on a horizontal shaker at room temperature. Then, a chemical fluorescence reaction was carried out. Image J Pro Plus 6.0 image analysis software was used to select the target protein band on the membrane after the chemical fluorescence reaction, and detected the protein gray value. β-actin was used as the internal reference for comparison, and the protein expression level was calculated.

### Elisa

On day 12 after surgery, collected serum from rats in each group or collected cell culture medium 12 h after OEC treatment, removed the kit and required strips, established a standard curve and standard 8 wells, added 100 μL of sample to each well, and set the eighth well as a blank control. 100 μL of serum or cultured medium was added to be tested to each well, and placed the reaction plate at 37°C for 2 h. Then rinse with washing solution and blot dry with filter paper. 50 μL of primary antibody was added to each well, and reacted for 1 h at 37°C. 100 μL of enzyme-labeled antibody working solution was added to each well, placed it at 37°C to react for 1 h, and then washed it. 100 μL of substrate reaction solution was added to each well, and reacted at 37°C in a dark place for 15 min. Then added 50 μL of stop solution to each well, and a photometer was used to measure absorbance at 450 nm. Four holes were randomly selected for testing, and the average of the results obtained was taken.

### Immunofluorescence

On day 12 after surgery, tissue sections or 24-well plates seeded with cells were rinsed three times in PBS, and goat serum was added to block for 1 h. P2X7R rabbit antibody (1:100), NSE rabbit antibody (1:100, Sigma-Aldrich), CD86 rabbit antibody (1:200, Sigma-Aldrich), peripherin rabbit antibody (1:500, Wuhan Sanying Co., Ltd.), and Neu rabbit antibody (1:200, Wuhan Sanying Co., Ltd.) were added and reacted at 37°C for 2 h, and washed with PBS. Goat anti-rabbit fluorescent secondary antibody (1:500, Jackson company) was added and incubated for 1 h in the dark, and washed with PBS. Then the tablets were sealed with a fluorescent sealing tablet, and the fluorescence of the labeled positive cells was observed under a fluorescent microscope. Four fields of view were randomly selected, and Image J Pro Plus 6.0 image analysis software was used to detect the percentage of labeled positive cells and fluorescence intensity in each field of view.

### H&E staining

On the 12th day after surgery, the tibial tissue was taken and made into tissue sections. Paraffin slices were baked, then dewaxed and hydrated. Placed the sections in hematoxylin aqueous solution and stained for 3 min, differentiated with hydrochloric acid ethanol differentiation solution for 15 s, washed with water, turn blue solution turned blue for 15 s, washed with water, stained with eosin for 3 min, washed with water, dehydrated, transparent, seal, and conduct microscopic examination.

### TRAP staining

Trap staining is a stain used to detect characteristic substances in bone tissue and bone cells, making osteoclasts red and the background green or blue. Tartrate-resistant acid phosphatase (Trap) is a marker enzyme of osteoclasts. It is specifically distributed in osteoclasts and is unique to osteoclasts. It is often used as an important marker for identifying osteoclasts. Under acidic conditions containing tartaric acid, trap can hydrolyze naphthol AS-BI phosphate, and the produced naphthol AS-BI immediately combines with fast red or hexaazo parafuchsin, forming an insoluble red dye at the active site of the enzyme. By observing the formation of the red dye, the activity of acid phosphatase can be indirectly understood, and the state of osteoclasts can be further identified and analyzed.

### Cell scratch test

Microglia (7 × 10^5^ cells, obtained from standard calculations of cells using an electronic automatic cell counter) were seeded in a six-well plate for culture. After the cells were spread throughout the well, a 10-µL gun tip was used to scratch, the scratched cells were washed with PBS, and OECs were added for co-culture for 24 h. The healing of the scratch was observed under the microscope at 0 and 24 h, respectively. Finally, the cell healing area before and after scratch healing was observed and measured through a microscope. Cell scratch healing rate (%) = (initial scratch area) − (scratch area after 24 h)/(initial scratch area) × 100%. Edge detection and quantitative analysis of cell scratches were performed using Image J Pro Plus 6.0 software, and the average area between cells was calculated by randomly selecting four horizontal lines.

### Cell migration experiment

Transwell migration 24-well plates were used to conduct cell migration tests. 200 µL of 1 × 10^5^cells (obtained from standard calculations of cells using an electronic automatic cell counter) were seeded into the chambers, 200 µL of OECs and their culture medium were added into the 24-well plate, and the chambers were placed into the wells for culture. After 24 h, the chamber was removed, the culture medium in the well was poured out, and 4% paraformaldehyde was added to fix the cells in the chamber for 15 min and washed with PBS. 400 µL of methyl violet staining solution was added to stain the cells in the chamber, washed with PBS, wiped off the liquid and excessed cells in the chamber with a cotton swab, and then placed the chamber under an inverted microscope for observation and photo counting. Four fields of view were randomly selected, and the migrating cells were located and counted using Image J Pro Plus 6.0 software.

### X-ray imaging

On the 12th day after intrathecal transplantation of OECs, the osteolytic state of the hind limbs was observed by X-ray photography. Anesthetized rats were placed in a prone position and exposed to X-rays at 45 kV for 2 s using an MX-20 Faxitron instrument (Itron company, USA). The osteolytic area of the hindlimbs was quantified using Image J software 1.52 (NIH, Bethesda, MD, USA).

### Methyl violet staining

The cell culture medium inoculated in the 24-well plate that was co-cultured with or without OECs was discarded and washed three times with PBS. 4% paraformaldehyde was added for fixation for 15 min and washed with PBS. Methyl violet staining solution was added for staining for 5 min and washed with PBS. The 24-well plate was placed under an inverted microscope and white light was used to observe the morphological changes of cells under culture and taken photos.

### Statistical methods

Statistical analysis was carried out using SPSS 20.0 software. The data were expressed as mean ± SD using Graphpad.prism. 6.x.crack-tsrh. Student’s *t*-test was used to compare between the two groups. One-way analysis of variance was used for multiple group comparisons. Two-way analysis of variance was used to compare differences over time and between treatment groups, followed by a least significant difference *post hoc* test. *P* < 0.05 was statistically significant.

## Results

### OEC culture results

**Figure 1.**
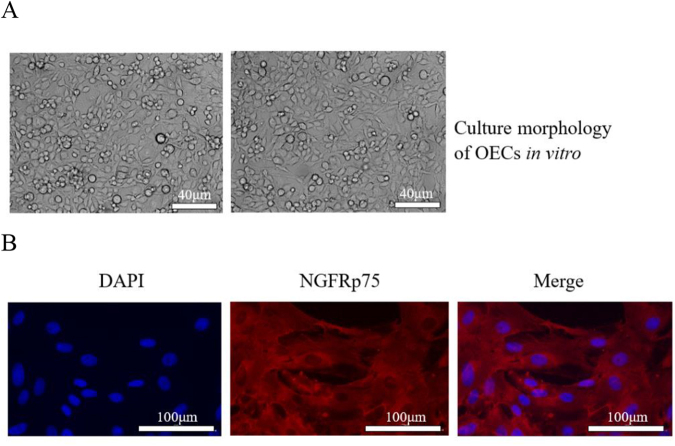
*In vitro* culture and identification results of OECs. (A) Morphology of OECs cultured *in vitro* under a microscope. Under the microscope, OECs are long, fusiform, and evenly distributed, bar = 40 μm. (B) Immunofluorescence identified the expression of NGFRp75 (a specific marker for OECs). NGFRp75 is uniformly expressed in cells. Bar = 100 μm.

Neonatal rat olfactory bulb tissue was taken, purified, and cultured *in vitro*, and the OECs showed long spindle shapes under white light, evenly distributed, and grew rapidly (Fig. 1A). Further immunofluorescence identification using the OEC-specific marker NGFRp75 showed positive staining and uniform distribution (Fig. 1B). These have laid a solid foundation for later OEC transplantation to explore the treatment of cancer pain.

### Pathological changes of rat tibia

**Figure 2.**
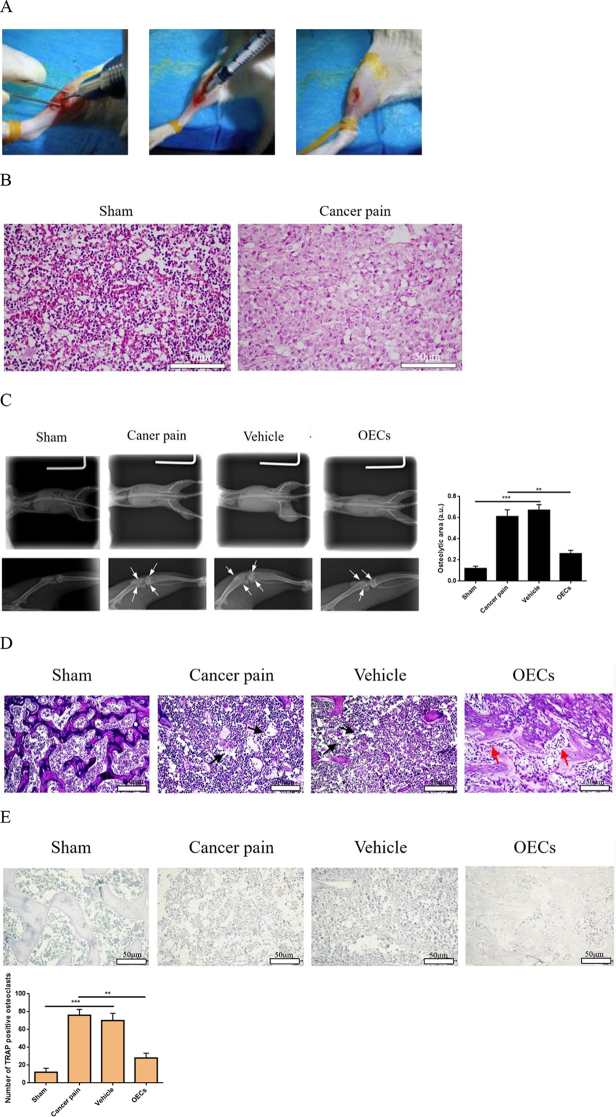
Changes of tibial bone before and after OEC transplantation. (A) The process of establishing a cancer pain model. (B) H&E staining was used to observe the pathological changes of tibial bone. Severe destruction of the trabecular bone structure in rat tibial tissue was observed in the model group, bar = 50 μm. (C) X-ray imaging observed the destruction and repair of tibial bone after OEC transplantation. Significant bone destruction and osteolysis were observed in rats in the cancer pain group and the vehicle group. However, after transplantation of OECs, bone destruction and osteolysis in the rat tibia were reduced (bone destruction and osteolytic lesions shown by arrows). (D) H&E staining was used to detect the tibia pathology of rats in each group, bar = 50 μm. The trabecular structure and bone of rats in the cancer pain group were severely damaged. However, after OEC transplantation, the damage to the tibial trabecular structure and bone destruction were significantly reduced (black arrows represent discontinuous bone trabeculae, and red arrows represent continuous bone trabeculae repaired after OEC transplantation). (E) TRAP staining was used to detect the bone destruction (osteoclast changes) of rats in each group, bar = 50 μm. More TRAP-positive osteoclasts were observed in the tumor–bone interface stained with TRAP in the cancer pain group and the vehicle group. However, the number of TRAP-positive osteoclasts at the tumor–bone interface was significantly reduced after OEC transplantation. Data are expressed as the mean ± SD. *N* = 6 per group (six rats were randomly selected from each group to X-ray the operated side and the changes in tibial bone were detected). One-way analysis of variance or Student’ *t*-test was used for inter-group comparisons, followed by *post hoc* test). ^**^P < 0.01, ^***^P < 0.001.

In order to understand the effect of OEC transplantation on bone destruction and repair of breast cancer after bone metastasis, a rat cancer pain model was first created (Fig. 2A). H&E staining was used to detect the changes in tibial bone. Compared with the sham group, the bone trabecular structure in the cancer pain group was severely damaged, and the bone marrow was filled with tumor cells (Fig. 2B). Day 12 after intrathecal injection, X-ray imaging assessed tibial bone destruction and repair. Figure 2C showed that the tibia of rats in the sham group had a normal shape, complete bone cortex continuity, and no bone destruction. Rats in the cancer pain group and the vehicle group showed obvious signs of clear bone destruction, interruption of bone cortex, and patchy shadows in cancellous bone. However, after transplantation of OECs, improved tibial bone cortex, reduced bone destruction, and continuous callus formation were observed in rats. Moreover, H&E and TRAP staining were used to detect the tibia pathology and bone destruction of rats in each group. The results showed that compared with the sham group, the rat bone trabecular structure in the cancer pain group was severely damaged and the bone marrow was filled with tumor cells. However, after OEC transplantation, it was found that the damage to the tibial bone trabecular structure was significantly relieved, and the number of osteoclasts was reduced (Fig. 2D,E). These data indicate that OEC transplantation may have a functional role in inhibiting bone destruction induced by tumor cells, inhibiting osteoclast activity and osteolysis, and promoting osteoblast activity. However, their specific and detailed mechanism of action is not yet very clear and requires further research and discussion in the future.

### Effects of OEC transplantation on pain behavior in rats with cancer pain

**Figure 3.**
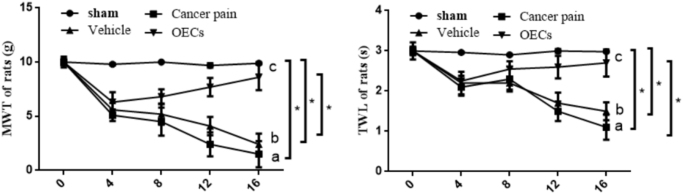
Effect of OEC transplantation on pain sensation in rats. The changes of MWT and TWL in rats in each group were measured by using behavioral methods at 0, 4, 8, 12, and 16 days after OEC transplantation. Compared with the sham group, the MWT and TWL of rats in the cancer pain group and the vehicle group were significantly reduced. Compared with the cancer pain group and the vehicle group, OEC transplantation could significantly increase the MWT and TWL in rats. Data are expressed as the mean ± SD, *n* = 4 per group (four rats were randomly selected for testing, excluding death, infection on the operated side, and complete paralysis of the right hind limb of the rats in each group). One-way analysis of variance or Student’s *t*-test was used for inter-group comparisons. Two-way analysis of variance was used to compare differences over time and between treatment groups, followed by *post hoc* test. ^a^*P* < 0.05 cancer pain vs sham, ^b^*P* < 0.05 vehicle vs sham, ^c^*P* < 0.05 OECs vs cancer pain or vehicle. *P < 0.05.

In order to understand the changes in pain-sensing behavior in rats with cancer pain after transplantation of OECs, behavioral methods were used to detect the changes in MWT and TWL in rats. The changes of pain sensation in rats in each group were measured by using behavioral methods on days 0, 4, 8, 12, and 16. The results showed that compared with the sham group, the MWT and TWL of rats in the cancer pain group and the vehicle group were significantly reduced. Compared with the cancer pain group and the vehicle group, the pain threshold of rats was significantly increased after OEC transplantation (Fig. 3). These data demonstrate that OEC transplantation can alleviate breast cancer-induced bone destruction and relieve pain.

### *OECs inhibit microglia activation by downregulating P2X7R expression* in vitro

In order to investigate whether OECs can mediate P2X7R on microglia activation *in vitro*, microglia were cultured for 24 h *in vitro*, and 10 μL of P2X7R activator BzATP was added to activate the microglia (no BzATP treatment was used as a control). Then, OECs were added to the activated microglia culture system for co-culture. Western blotting was used to test the changes in P2X7R protein expression in microglia after OEC treatment. The results showed that P2X7R protein expression level in microglia significantly increased after BzATP treatment, while the OEC co-culture showed that P2X7R protein expression level in microglia decreased (Fig. 4A). The changes in fluorescence intensity of P2X7R-labeled positive cells were further detected by immunofluorescence. The results showed that BzATP significantly increased the fluorescence intensity of P2X7R-labeled positive cells, while the fluorescence intensity of P2X7R-labeled positive cells decreased after co-culture of OECs (Fig. 4B).

**Figure 4.**
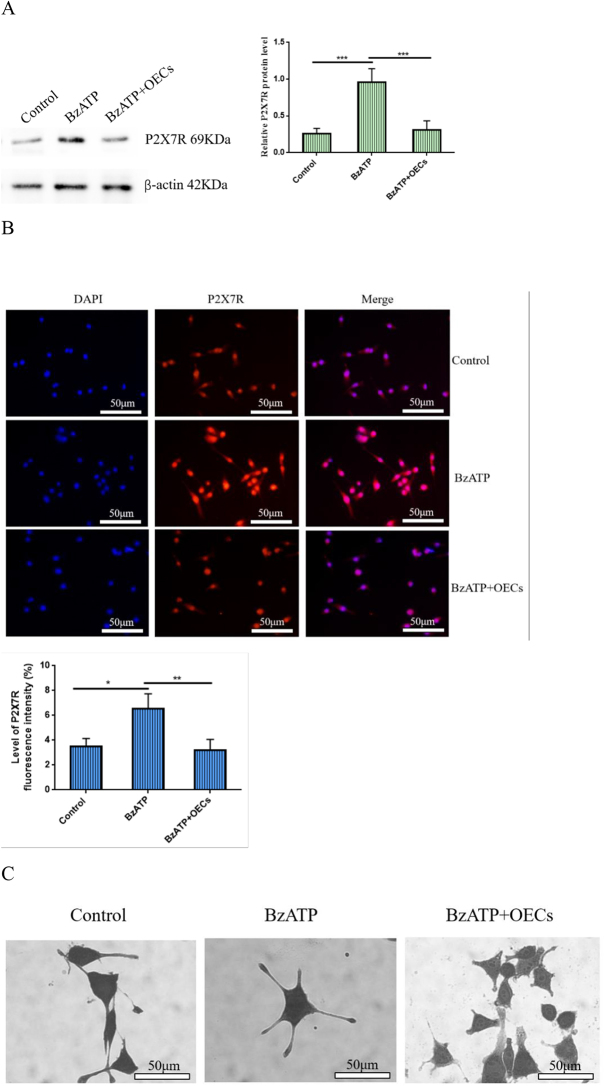
Effect of OECs on P2X7R-mediated microglia activation *in vitro.* (A) Western blotting was used to detect the changes of P2X7R protein expression levels in microglia. P2X7R agonist BzATP increased P2X7R protein expression in microglia, while OECs decreased P2X7R expression in microglia. (B) The change in fluorescence intensity of P2X7R-labeled positive cells was detected by immunofluorescence. Under the activation of microglia by BzATP, OECs could significantly reduce the fluorescence intensity of P2X7R-labeled positive cells, bar = 50 μm. (C) Morphological changes of microglia were observed with methyl violet staining, bar = 50 μm. (D) Transwell migration assay was used to test the changes in migration of microglia, bar = 40 μm. (E) Cell scratching assay was used to test the changes in microglia migration ability. OECs inhibited the BzATP-induced migration and movement of microglia, bar = 20 μm. (F) qRT-PCR was used to detect the changes in mRNA expression levels of M1 microglia (CD86) and M2 marker (CD163). BzATP increased the mRNA expression level of M1 marker CD86 in microglia and decreased the mRNA expression level of M2 marker CD163. However, this phenomenon was reversed after treatment with OECs. (G) The changes in CD86 fluorescence intensity were detected by immunofluorescence, bar = 50 μm. (H) Elisa measured the changes in the concentration level of inflammatory cytokines IL-1β and IL-18. After treatment with OECs, the concentration level of IL-1β and IL-18 released after BzATP-induced activation of microglia could be significantly reduced. Data are expressed as the mean ± SD. *N* = 4 (in each group, four samples were randomly selected for testing). One-way analysis of variance or Student’s *t*-test was used for inter-group comparisons, followed by *post hoc* test. ^*^P < 0.05; **P < 0.01; ***P < 0.001.

In order to understand the changes in P2X7R-mediated microglia activation after co-culture of OECs, morphological changes of microglia were observed by using methyl violet staining. The results showed that microglia were significantly activated after BzATP treatment, with enlarged cell bodies and increased and lengthened peripheral processes. However, after co-culture of OECs, microglia became smaller in shape, with reduced cell bodies and processes (Fig. 4C). Moreover, cell scratching and cell migration experiments verified the influence of OECs on microglia. The results showed that BzATP promoted the migration and mobility of microglia, while the migration and mobility of microglia decreased after OEC treatment (Fig. 4D,E).

**Figure 4.**
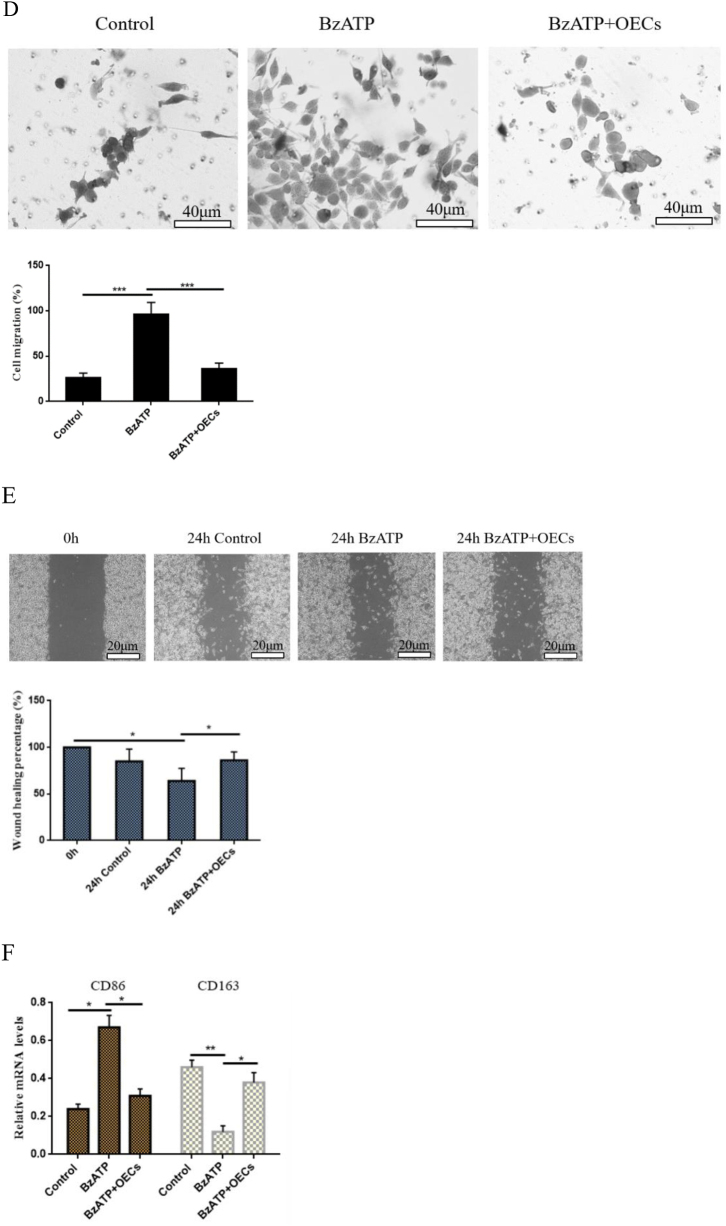

To further understand the polarization status of microglia, qRT-PCR was used to detect the changes in mRNA expression levels of M1 microglia (CD86)/M2 marker (CD163)^[28,29]^. Figure 4F showed that P2X7R activator BzATP significantly increased the expression level of CD86 mRNA and decreased the expression level of CD163 mRNA. Immunofluorescence showed that BzATP increased the fluorescent intensity level of CD86-labeled cells, while the opposite result was obtained after OEC treatment (Fig. 4G).

**Figure 4.**
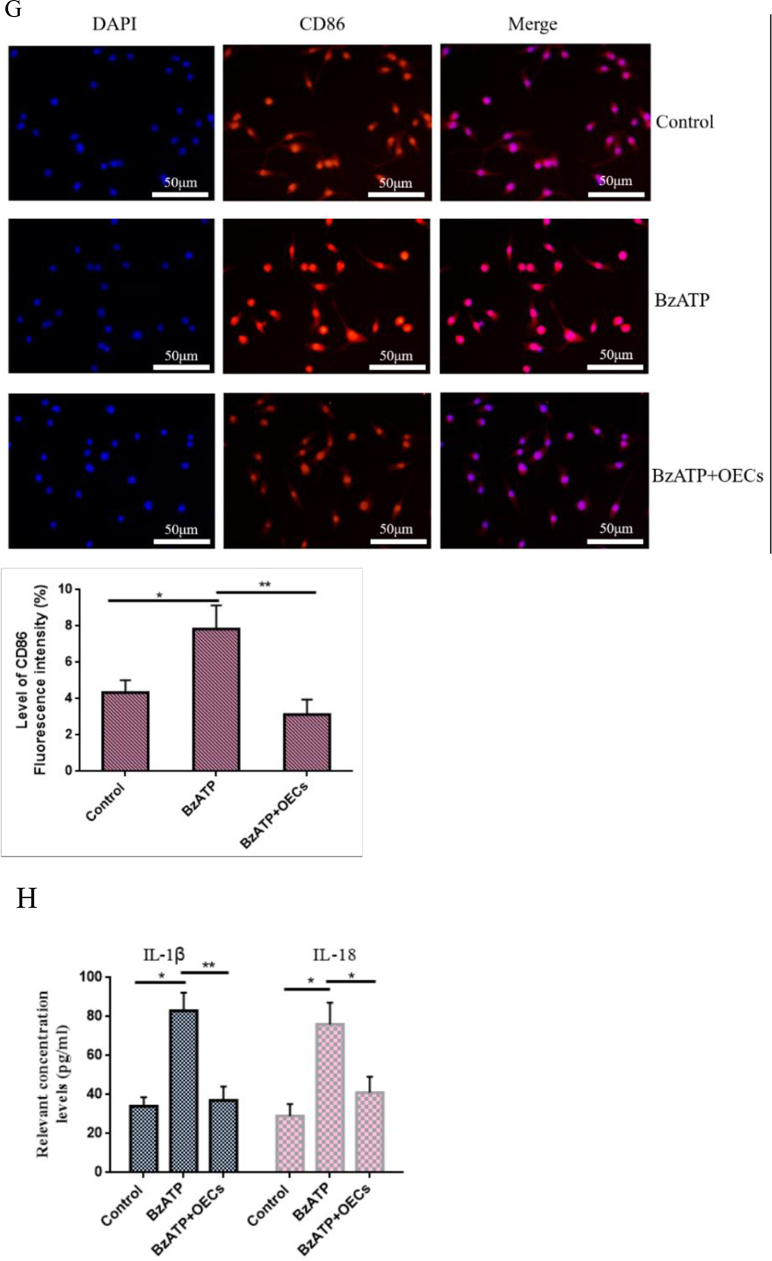

Studies have shown that P2X7R-mediated microglia activation can release inflammatory cytokines. Therefore, we used Elisa to detect the changes in the levels of pro-inflammatory factors IL-18 and IL-1β in the culture medium of the co-culture system. It was found that BzATP increased the concentration levels of IL-18 and IL-1β in the medium, while co-culture of OECs decreased the concentration levels of IL-18 and IL-1β (Fig. 4H). These data suggest that OECs can inhibit microglia activation and inflammatory cytokine release by downregulating P2X7R expression.

### Effect of OEC transplantation on neuron growth

Studies have shown that P2X7R activation can activate microglia to mediate the release of inflammatory cytokines, which have toxic effects on neurons. Therefore, we next investigated whether OECs have a functional role in protecting neurons. Microglia were cultured *in vitro*, and the P2X7R agonist BzATP 10 μL was added to the microglia culture system for 24 h to activate the microglia. Then, the microglia culture medium was added to the neuron co-culture system and continued co-culture for 24 h (no activated microglia medium treatment was used as a control). The growth changes of neurons were observed by using methyl violet staining. The results showed that BzATP induced microglia activation and significantly inhibited the morphological changes and axon growth of neurons, shrinking the cell bodies and shortening the processes. Co-culture of OECs significantly increased the survival of neurons and promoted the growth and extension of their processes (Fig. 5A). Immunofluorescence was used to detect the fluorescence intensity of neuron-specific marker NSE. The results showed that BzATP reduced the fluorescence intensity of NSE-labeled cells, while the fluorescence intensity of NSE-labeled positive cells increased after OEC treatment (Fig. 5B). Moreover, CCK-8 was used to measure the survival of neurons, and the results showed that treatment with OECs increased the survival of neurons (Fig. 5C).

**Figure 5.**
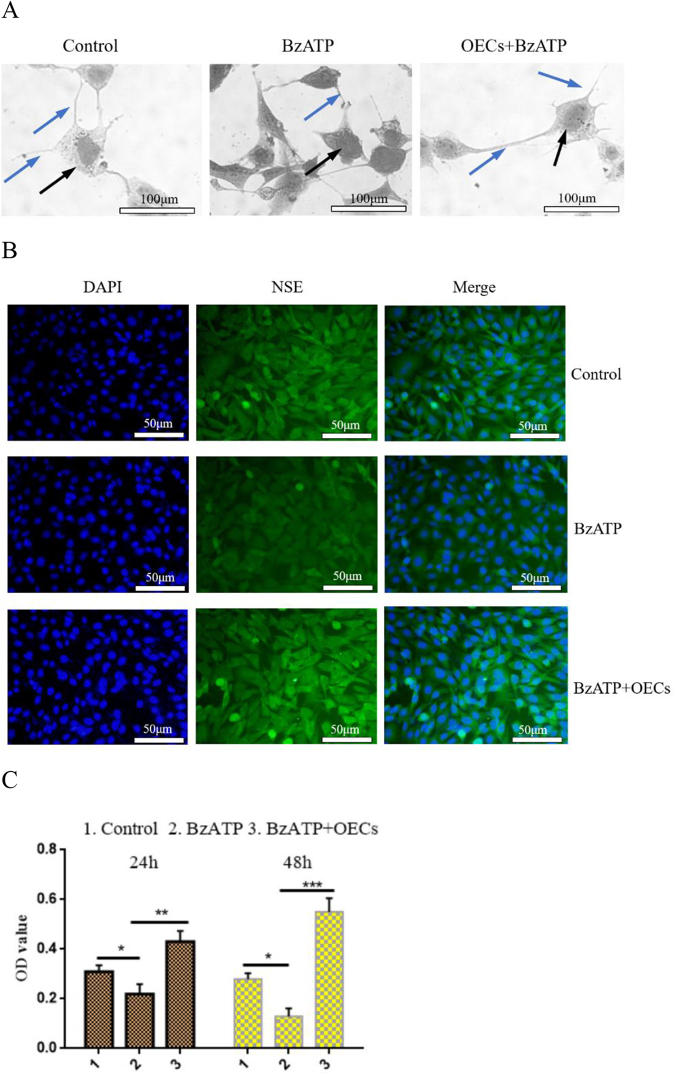
Effect of OECs on neuron survival and neuroprotection. (A) Morphological changes of neurons cultured *in vitro* were observed with methyl violet staining. BzATP-induced activation of microglia significantly inhibited axonal growth of neurons, while OEC treatment promoted neuronal survival and axonal extension, bar = 100 μm. (B) The changes in fluorescence intensity of neuron marker NSE were detected by immunofluorescence, bar = 50 μm. (C) CCK-8 was used to measure the neuron survival. P2X7R agonist BzATP activated microglia and co-cultured with neurons inhibited neuron survival, while OEC treatment protected neuron survival. (D) After transplantation of OECs *in vivo*, the changes in fluorescence intensity of Neu, a neuron marker in spinal cord tissue, were detected by immunofluorescence. The fluorescence intensity of Neu-labeled neurons decreased significantly in the cancer pain group and the vehicle group, while the fluorescence intensity of Neu-labeled neurons increased after OEC transplantation, bar = 50 μm. (E) Immunofluorescence was used to analyze the changes in axon growth of peripherin. Compared with the cancer pain group and the vehicle group, the percentage of peripherin-labeled positive cells in the OEC transplantation group was lower, bar = 100 μm. Data are expressed as the mean ± SD, *n* = 4 per group (four samples were randomly selected for testing, excluding infection on the operated side, ischemic necrosis of the dorsal root ganglia and spinal cord tissues, and destruction of dorsal root ganglia and spinal cord tissues during sampling and the experiment). One-way analysis of variance or Student’s *t*-test was used for inter-group comparisons, followed by *post hoc* test. ^*^P < 0.05; **P < 0.01; ***P < 0.001.

OECs were transplanted into rats with cancer pain. The fluorescence changes of Neu-labeled neurons in spinal cord tissue were detected by taking the spinal cord tissue of rats. The results showed that the fluorescence intensity of Neu-labeled neurons in spinal cord tissue in the cancer pain group and the vehicle group was significantly reduced, while the fluorescence intensity of Neu-labeled neurons increased significantly after OEC transplantation (Fig. 5D). Moreover, immunofluorescence was further used to analyze the growth of peripherin axons in dorsal root ganglia. Figure 5D showed that compared with the sham group, the percentage of peripherin-labeled positive cells in the cancer pain group and the vehicle group increased significantly, while the percentage of peripherin-labeled positive cells decreased after OEC treatment (Fig. 5E). These data suggest that OECs can downregulate P2X7R-mediated microglia activation and play a neuroprotective role.

**Figure 5.**
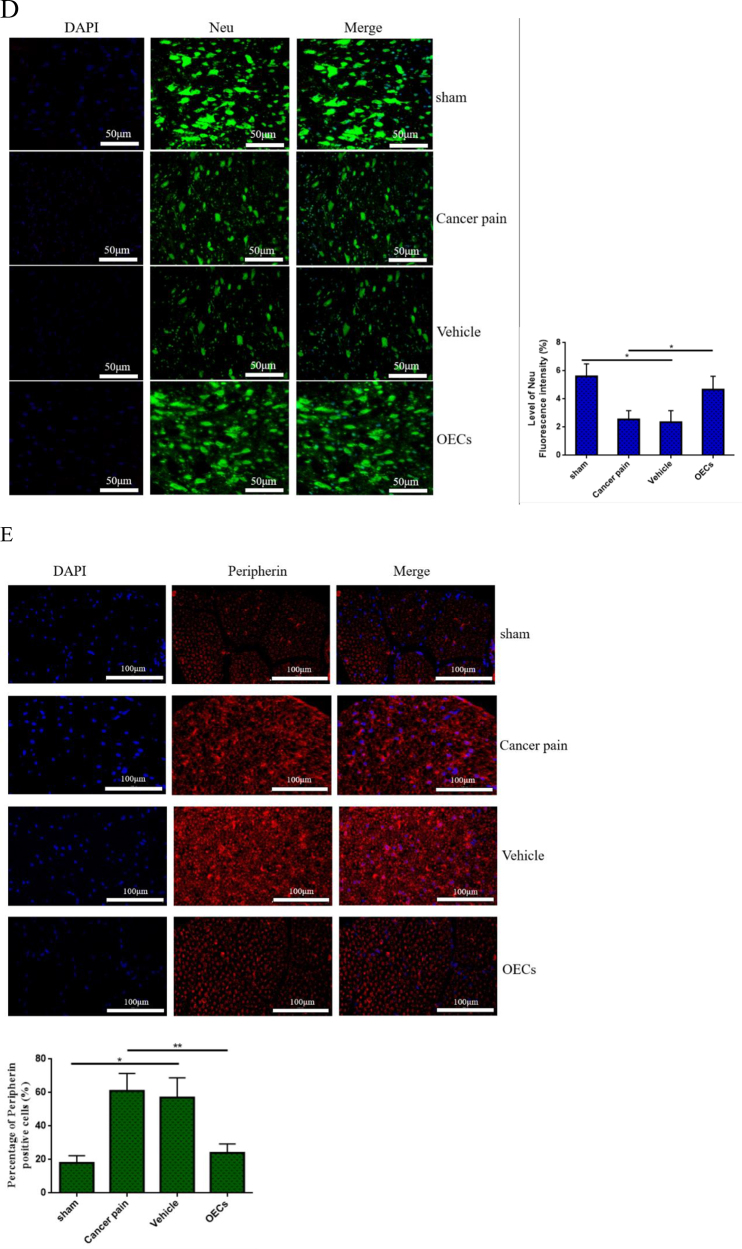

### OEC transplantation downregulates P2X7R expression, inhibits NLRP3 activation, and improves neuroinflammatory response

To understand the role of OEC transplantation on P2X7R-mediated NLRP3 in cancer pain, on the 12th day after the transplantation of OECs, spinal cord tissues of rats in each group were taken, and the expression of P2X7R in spinal cord tissues was detected by using immunohistochemical staining. The results showed that compared with the sham group, the number of P2X7R-labeled positive cells in the cancer pain group increased significantly and the staining was deeper. While the P2X7R-labeled positive cells in the vehicle group did not change significantly. However, compared with the cancer pain group or the vehicle group, the number of P2X7R-labeled positive cells and staining were significantly reduced after OEC transplantation (Fig. 6A). Moreover, the expression level of P2X7R in spinal cord tissue was detected by immunofluorescence, and consistent results were obtained. The percentage of P2X7R-labeled positive cells and fluorescence intensity were significantly reduced after OEC transplantation (Fig. 6B). Western blotting showed that compared with the cancer pain group or the vehicle group, the expression level of P2X7R protein was significantly reduced after OEC transplantation (Fig. 6C). In addition, the co-localization and expression of microglial markers IBA-1 and P2X7R in spinal cord tissue were further detected by immunofluorescence double staining. The results showed that IBA-1 and P2X7R were co-expressed in microglia. Compared with the sham group, the cancer pain group and the vehicle group showed that the fluorescence intensity of IBA-1- and P2X7R-labeled positive cells increased, while the fluorescence intensity of IBA-1- and P2X7R-labeled positive cells decreased after OEC transplantation (Fig. 6D).

**Figure 6.**
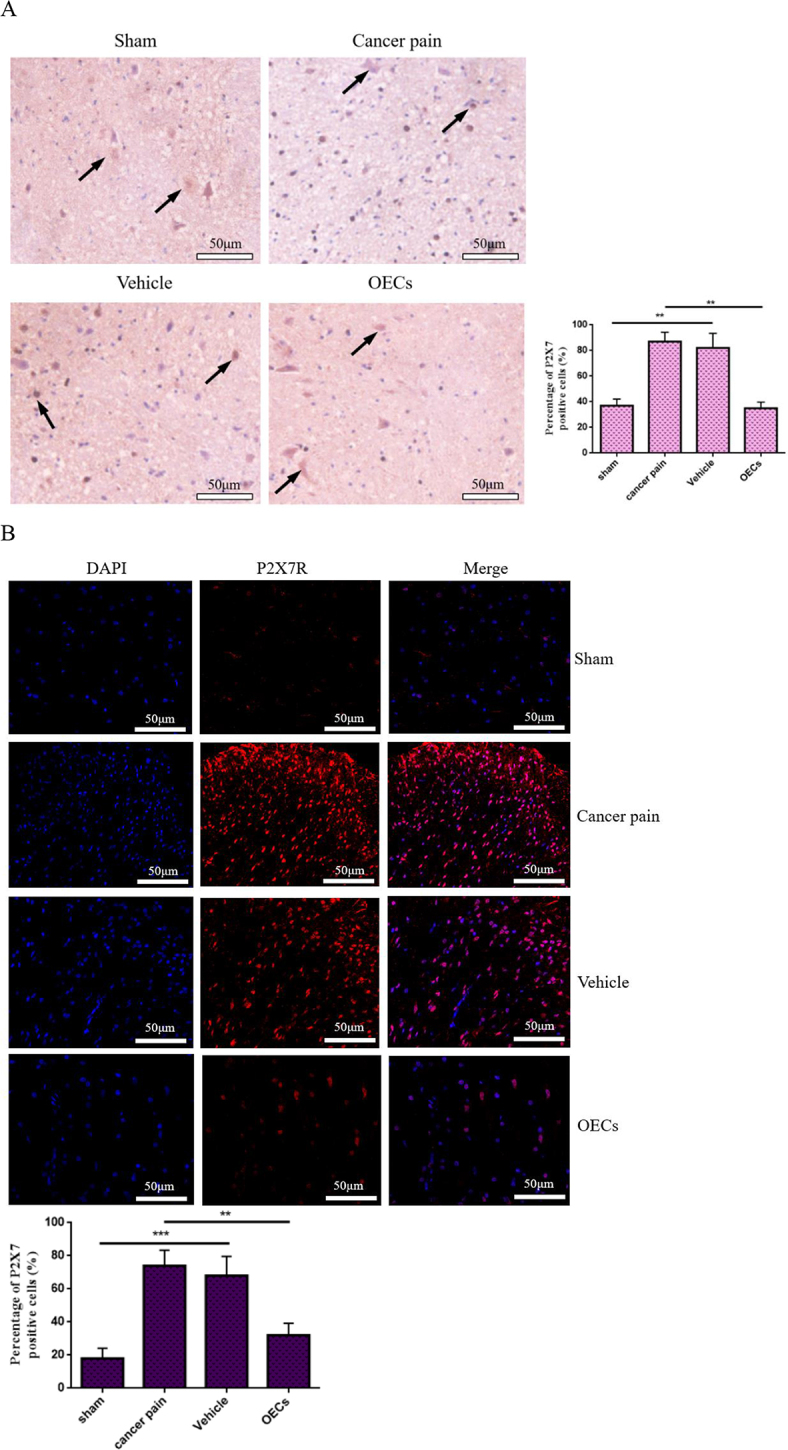
Effect of OEC transplantation on P2X7R-mediated neuroinflammatory response. (A) Immunohistochemical staining was used to detect the expression of P2X7R in spinal cord tissue. Compared with the cancer pain group and the vehicle group, the percentage of P2X7R-labeled positive cells in spinal cord tissue after OEC transplantation was significantly reduced, bar = 50 μm. (B) Immunofluorescence was used to detect P2X7R expression in spinal cord tissue. In the cancer pain group and the vehicle group, the percentage of P2X7R-labeled positive cells increased significantly, while the percentage of P2X7R-labeled positive cells decreased after OEC transplantation, bar = 50 μm. (C) Co-expression of microglial markers IBA-1 and P2X7R in spinal cord tissue was detected by immunofluorescence. IBA-1 and P2X7R were co-expressed in microglia. Compared with the cancer pain group and the vehicle group, the fluorescence intensity of IBA-1- and P2X7R-labeled positive cells in spinal cord tissue after OEC transplantation was significantly reduced, bar = 100 μm. (D) Western blotting was used to measure the P2X7R protein expression in spinal cord tissue. (E) Western blotting was used to measure NLRP3/ASC/caspase-1 protein expression in spinal cord tissue. After OEC treatment, NLRP3/ASC/caspase-1 protein expression levels decreased in spinal cord tissue. (F) Elisa was used to detect the concentration changes of IL-1β and IL-18 in rat serum. Compared with the cancer pain group and the vehicle group, the concentration levels of IL-1β and IL-18 in the serum of rats after OEC transplantation were significantly reduced. Data are expressed as the mean ± SD, *n* = 4 per group (spinal cord tissues of four rats in each group were randomly selected for testing, excluding death and infection on the operated side of the rats, spinal cord tissue injury during the sampling process, and tissue destruction during the experimental process). One-way analysis of variance or Student’s *t*-test was used for inter-group comparisons, followed by *post hoc* test. ^*^P < 0.05; **P < 0.01; ***P < 0.001.

**Figure 6.**
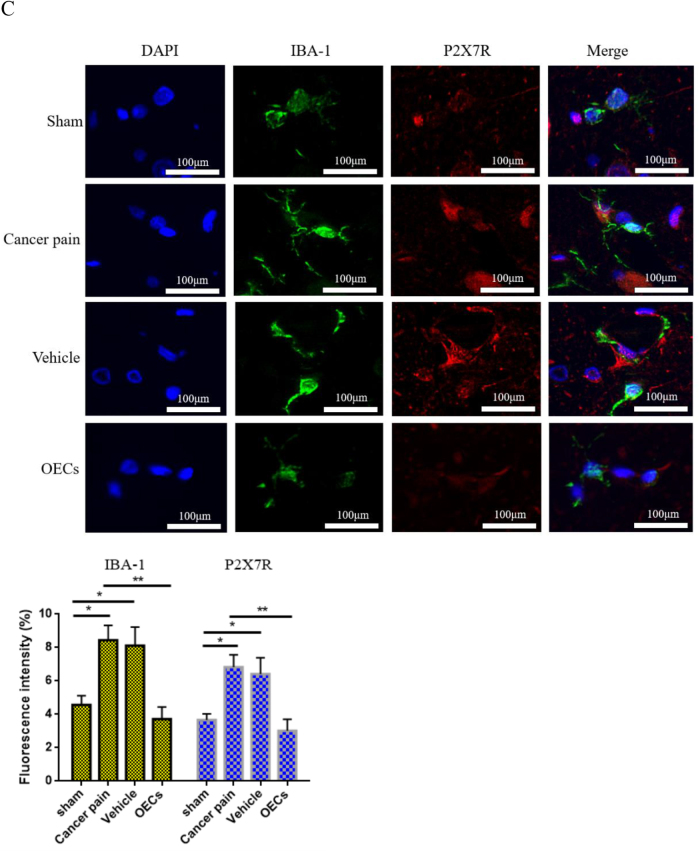

**Figure 6.**
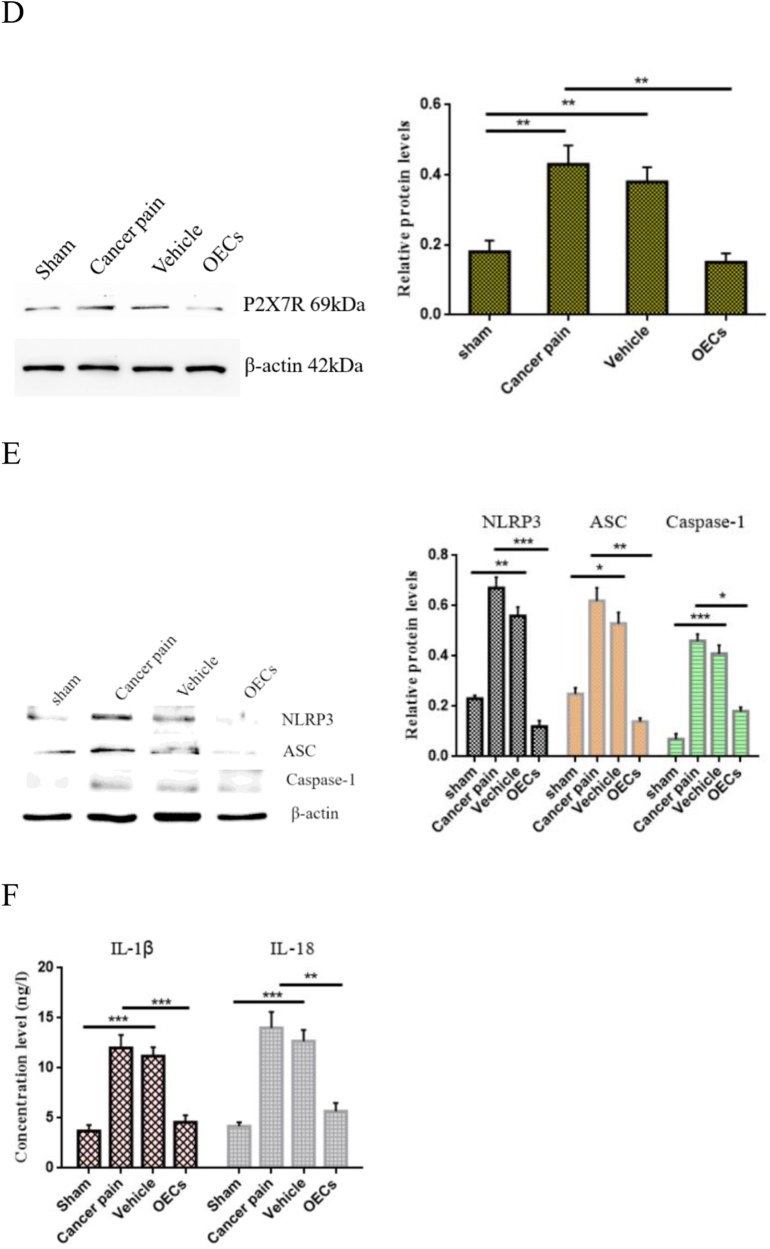

Activation of P2X7R mediates NLRP3 inflammasome activation and subsequent cytokine release. Therefore, the changes in NLRP3/ASC/caspase-1 expression levels in spinal cord tissue were next detected by Western blotting. Figure 6E showed that compared with the sham group, the levels of NLRP3, ASC, and Caspase-1 protein were significantly increased in the cancer pain group and the vehicle group, but the levels of NLRP3, ASC, and Caspase-1 protein were significantly decreased after OEC treatment. Moreover, the changes in IL-1β and IL-18 concentrations in rat serum were further detected by Elisa. The results showed that compared with the cancer pain and vehicle groups, the concentration levels of IL-1β and IL-18 were significantly reduced after OEC transplantation (Fig. 6F). These studies have shown that OEC transplantation can reduce neuroinflammatory responses and play a neuroprotective and pain-relieving functional role by downregulating the expression of P2X7R.

## Discussion

Cancer pain belongs to the chronic type of pain, which is caused by the direct or indirect exposure of the nervous system to harmful stimuli, resulting in physical dysfunction, including sensory and motor functions. Tumor cells can migrate remotely along the nerve fibers or invade surrounding tissues and organs, lead to sensitization of primary receptors, and enhance sensory signal transmission^[1,30]^. The manifestations of cancer pain are more intense and lasting than other pain types, seriously affecting the patient’s physical and mental health and reducing the patient’s quality of life in the later stages. Opioids are often used clinically to relieve pain in patients, but approximately 32% of patients with cancer pain are not adequately treated. Although for patients who respond to powerful opioids, long-term use of opioids can cause many side effects, such as bowel movements, tolerance, addiction, and use of large doses^[31]^. In addition, drugs have no fundamental therapeutic effect on injured nerves, and there is no possibility of rebuilding neural network function to achieve long-term therapeutic effects. This is also a shortcoming of current drug treatment. Therefore, it is particularly important to explore a promising method to effectively treat cancer pain.

Cancer pain involves multi-factorial and multi-molecular-based pathological processes. Therefore, it is of great significance to find molecular targets closely related to cancer pain for effective targeted treatment. Studies have confirmed that P2X7R plays a key functional role as a pain-related molecule in the transmission of pain information^[32,33]^. There is growing evidence that activation of spinal cord microglia contributes to the development and maintenance of inflammation and neuropathic pain^[15,29]^. Microglia activation can enhance synaptic transmission in spinal cord nociceptive neurons by producing pro-inflammatory cytokines (IL-18), inducing cancer pain^[15]^. P2X7R is widely expressed in the nervous system, mainly expressed in microglia, and is closely related to the stability and maintenance of nervous system function^[8,34]^. After the nervous system encounters a noxious stimulus, the expression of P2X7R increases significantly, activates the microglia-mediated neuroinflammatory response, amplifies nerve tissue damage, and induces pain generation^[35,36]^. Studies have shown that P2X7R inhibitor BBG inhibits the expression of spinal cord NF-kB p-p65, NLRP3, and downstream cytokine IL-1β to effectively inhibit bone cancer pain^[37]^. P2X7R siRNA reduces microglial activity, nuclear transport of NF-kB, and synthesis of NLRP3 and IL-1β in BV2 cells^[37]^. Bone cancer pain upregulates P2X7R, phosphorylates p38, and increases IL-18 levels in spinal cord microglia, while inhibition of the spinal cord P2X7R/p-38/IL-18 pathway reduces pain in advanced bone cancer and suppresses neuron overactivity^[15]^. Studies have shown that Gallic acid reduces the expression of P2X7R and p-ERG1/2 in the hippocampus, spinal cord, and DRG of rats, reduces serum IL-1β and TNF-α concentrations in rats, increases IL-10 levels, and relieves pain and depression^[38]^. In this study, we also found that P2X7R activator BzATP could activate microglia, increase NLRP3 expression and release of inflammatory cytokines IL-1β and IL-18, and have toxic effects on neurons. The results we obtained are consistent with those obtained in previously published studies. These studies have revealed that P2X7R participates in pain progression by activating microglia-mediated release of inflammatory cytokines. Therefore, targeted downregulation of P2X7R expression and inhibition of the inflammatory response mediated by microglia activation may become another potential target approach for pain treatment.

Researchers have explored many ways to promote the repair of damaged nerves and relieve pain, but in recent years, alternative strategies for cell transplantation have entered people’s horizons and aroused widespread interest. Cell replacement therapy can promote the repair of injured nerves, can rebuild neural network functions, can improve the local unfavorable microenvironment of nerve injury, can promote axon regeneration and re-myelination, and has been widely recognized in the field of nerve injury regeneration^[17,39]^. OECs are a special type of glial cells that survive and self-renew throughout life in the central system. They have a high survival rate *in vitro* culture. They also secrete a variety of neurotrophic factors, which are conducive to axon regeneration and damaged nerve repair. In addition, OECs also have anti-inflammatory and immunomodulatory properties, which can improve the inflammatory microenvironment of nerve injury, and are conducive to damaged nerve repair and pain relief^[19,21,40,41]^. Studies have found that OEC transplantation promotes nerve regeneration and the restoration of larynx tongue and voice in dogs with recurrent laryngeal nerve injury^[42]^. OEC transplantation can reduce the levels of P2X7R, NLRP3, NF-kB, IL-1β, and IL-18 in rats, reduce neuroinflammatory response, and relieve pain induced by trigeminal nerve injury in rats^[43]^. We previously studied that microencapsulated OECs transplanted into the sciatic nerve injury in rats could reduce the expression of P2X7R in spinal cord tissue and relieve hyperalgesia in rats^[21]^. In research on other types of pain, such as neuropathic pain, OEC transplantation has been revealed to have a functional role in alleviating pain. However, it is unknown whether OEC transplantation has an impact on cancer pain. Therefore, in this study, we used a previously established animal model of cancer pain with bone metastases from breast cancer^[44]^ and transplanted the OECs intrathecally into the rats with cancer pain. The results showed that OEC transplantation could inhibit the bone destruction caused by bone metastasis of breast cancer, downregulate the expression of P2X7R, inhibit the activation of NLRP3 inflammasome of microglia, and reduce the release of inflammatory cytokines, thus alleviating the pain behavior of cancer pain rats. Our results reveal that OECs have therapeutic effects in cancer pain, further confirming their functional role in relieving pain.

The experimental data obtained earlier suggest that OEC transplantation has a therapeutic functional effect in alleviating cancer pain. The possible mechanism for their analgesic effect is to downregulate the expression of P2X7R, inhibit the neuroinflammatory response mediated by microglia activation, and play a neuroprotective role, thereby achieving an analgesic therapeutic effect. However, this experiment has certain limitations. For example, P2X7R antagonists/antagonists were not used in *in vivo* experiments to further verify the regulation of P2X7R and subsequent signal transduction pathways by OECs, and how OECs interact with P2X7/NLRP3 signals upstream and downstream, which also provides a direction for our in-depth mechanism research. The detailed molecular mechanism by which OECs exert a therapeutic effect in the pathological mechanism of cancer pain needs to be further studied and explored in the future. In addition, this experiment also has limitations in animal and model construction. Female and male rats were used in this study. Gender differences may have an impact on rat behavioral results. In the later stage, animal selection needs to unify the gender of rats to avoid possible errors caused by gender differences. Although the breast cancer bone metastasis model is the most studied and used classic animal model of bone metastasis, this model has limitations. There is a mismatch between cell lines and clinical heterogeneity, and the tendency to bone metastasis may vary depending on cell subtypes. The application of transgenic mouse models (such as the MMTV-PyMT spontaneous breast cancer model) or using a humanized mouse model to simulate human tumor cell bone metastasis models may have better stability.

At present, most of the application of OECs in pain treatment is based on basic research, but there are many challenges in clinical application and transformation. First, the therapeutic effect of OEC transplantation in animal models has been successful and has achieved exciting results, but the success of animal models should not be directly applied to clinical transformation, which is related to the huge physiological differences and biological characteristics between humans and animals. The functional mechanism of OECs should be comprehensively and in detail in basic research, and clinical standardized treatment plans and implementation plans should be gradually established to better transition to the stage of clinical application exploration. Second, the standardized *in vitro* culture system for OECs is still imperfect. *In vitro* cell culture may be affected by the internal or external environment. For example, cell contamination may lead to unstable cell functions and cell abnormalities, which may lead to adverse results during clinical transformation. Moreover, the optimal time for cell transplantation and the dose of cells used currently lack consistency. These problems can lead to differences in the therapeutic effects of transplanted cells and even treatment failure. Third, there are challenges in transplantation technology and immune rejection. Currently, routinely used techniques include venous transplantation and intrathecal transplantation, which can be performed well by surgeons. However, for example, intrathecal transplantation requires additional surgical procedures, which can increase the risk of additional injury and infection. OECs are transplanted into the body can also be subject to immune rejection, which limits their ability to exert therapeutic effects. These challenges and problems have hindered the extensive development and transformation of OECs in clinical applications, but these problems will eventually be solved as researchers continue to explore in the future. In short, OECs are a promising candidate cell for the treatment of cancer pain, providing some new data to support the future treatment of cancer pain.

## Data Availability

All data generated or analyzed during this study are included in this article. And we have not used other data that have already been published. All the data presented in this article are original results derived from this study.
